# Increased mitochondrial fission of glomerular podocytes in diabetic nephropathy

**DOI:** 10.1530/EC-19-0234

**Published:** 2019-07-26

**Authors:** Yiqiong Ma, Zhaowei Chen, Yu Tao, Jili Zhu, Hongxia Yang, Wei Liang, Guohua Ding

**Affiliations:** 1 Division of Nephrology, Renmin Hospital of Wuhan University, Wuhan, Hubei, People’s Republic of China

**Keywords:** diabetes, metabolism

## Abstract

**Aims:**

Previous studies showed that abnormal mitochondrial structure and function were involved in the pathological process of diabetic nephropathy (DN). The dynamic mitochondrial processes, including fusion and fission, maintain the mass and quantity of mitochondria. Podocyte injury is a critical factor in the development and progression of DN. The present study evaluated the mitochondrial fission of podocytes in patients with DN.

**Methods:**

We recruited 31 patients with biopsy-confirmed DN. A quantitative analysis of the mitochondrial morphology was conducted with electron microscopy using a computer-assisted morphometric analysis application to calculate the aspect ratio values. Immunofluorescence assays were used to evaluate protein colocalization in the glomeruli of patients.

**Results:**

The urine protein level was significantly increased in DN patients compared to non-DN patients (*P* < 0.001), and the mitochondria in the podocytes from DN patients were more fragmentated than those from patients without DN. The mitochondrial aspect ratio values were negatively correlated with the proteinuria levels (*r* = −0.574, *P* = 0.01), and multiple regression analysis verified that the mitochondrial aspect ratio was significantly and independently associated with the urine protein level (β = −0.519, *P* = 0.007). In addition, Drp1, a mitochondrial fission factor, preferentially combines with AKAP1, which is located in the mitochondrial membrane.

**Conclusions:**

In the podocytes of DN patients, mitochondrial fragmentation was increased, and mitochondrial aspect ratio values were correlated with the proteinuria levels. The AKAP1-Drp1 pathway may contribute to mitochondrial fission in the pathogenesis of DN.

## Introduction

As a severe type of diabetic microangiopathy, diabetic nephropathy (DN) is considered the most common cause of end-stage renal disease (ESRD) worldwide (1). In China, there are 113.9 million people with diabetes, and the estimated number of patients with chronic kidney disease (CKD) related to diabetes is 24.3 million, which accounts for 21.3% of all individuals with CKD (2). Hence, DN has become the leading cause of CKD in China.

Persistent proteinuria, which indicates podocyte injury, is an important clinical feature of DN. Podocytes, also known as glomerular visceral epithelial cells, are located outside the glomerular basement membrane. The finger-like foot processes of podocytes closely intertwine to form the slit diaphragm (SD), which constitutes the most important component of the glomerular filtration barrier (3). With complex structural functions, podocytes play a key role in many renal diseases, and their injury/loss is considered to be a vital step in the progression of DN (4, 5), although the underlying molecular mechanism remains unclear.

Previous studies have shown that abnormalities in mitochondrial structure and function are involved in the pathogenesis of DN (6, 7). High glucose induction leads to abnormal mitochondrial biosynthesis, which causes a reduction in ATP synthesis and an increase in the generation of reactive oxygen species (ROS) (8, 9); therefore, mitochondrial dysfunction occurs before proteinuria and renal pathology (10). Furthermore, mitochondria are considered dynamic organelles that periodically divide (fission) and fuse (fusion) (11). These dynamic processes maintain stable mitochondrial mass and quantity (12). It was reported that hyperglycaemia results in increased expression of mitochondrial fission protein and decreased expression of mitochondrial fusion protein in renal tissue (10, 12). Podocyte-specific deletion of dynamin-related protein 1 (Drp1), which is a key factor in mitochondrial fission, mitigates the progression of DN (13). Our previous studies confirmed that high levels of glucose cause mitochondrial ROS production and increases the podocyte apoptosis rate (14, 15). However, whether mitochondrial dynamics contribute to the development and progression of DN in patients has not yet been fully elucidated. Thus, to assess the role of mitochondrial dynamics in the pathological progression of DN, we collected renal biopsies from DN patients and analysed the morphological changes in mitochondria in podocytes. The results supported the close association of mitochondrial dynamics and DN.

## Materials and methods

### Study population

Thirty-one eligible patients with renal biopsy-confirmed DN were selected in the Division of Nephrology, Renmin Hospital of Wuhan University from 2016 to April 2018. The control group included six patients with renal neoplasm, and normal kidney tissues were obtained from these patients by nephrectomy. The study protocol was approved by the Ethics Committee of Renmin Hospital of Wuhan University. All experiments were performed in accordance with the approved guidelines of Wuhan University. The study complied with the Declaration of Helsinki. Written informed consent was obtained from the patients for the publication of this study and any accompanying images.

### Mitochondrial morphology analysis

Digital images of the mitochondria were obtained using transmission electron microscopy (HITACHI, Japan). A quantitative analysis of the mitochondrial morphology was conducted using a computer-assisted morphometric application to calculate the aspect ratio values (16). Three podocytes were observed in each specimen. The acquired images of the mitochondria were analysed using ImageJ software. The aspect ratio values were derived from the lengths of the major and minor axes, and the average aspect ratio values were calculated for statistical analysis. The value 1 indicates a perfect circle. As mitochondria elongate and become more elliptical, the aspect ratio values increase.

### Immunofluorescence assay

Frozen kidney sections were blocked with 5% bovine serum albumin (BSA) for 30 min at 37°C. The sections were incubated with a mixture of primary antibodies (AKAP1 rabbit monoclonal antibody, 1:100, Cell Signaling Technology; Drp1 rabbit monoclonal antibody, 1:100, Abcam) overnight at 4°C. FITC/TRITC-conjugated IgG was used as a secondary antibody and was incubated with the sections at 37°C for 90 min in the dark. All microscopic images were recorded using a confocal microscope (Olympus).

### Statistical analysis

The data were expressed as the mean ± standard deviation, and the statistical analyses were performed using SPSS, version 19.0 (Chicago). Independent *t*-tests were performed to compare the variables between the DN and non-DN groups. Pearson’s correlations were used to characterize the associations between various characteristics and the mitochondrial aspect ratio value. Multiple linear regression analysis was used to evaluate the contribution of each confounding factor to the mitochondrial aspect ratio value. The results were considered statistically significant at *P* < 0.05.

## Results

### Clinical characteristics

Thirty-one patients with DN were enrolled in this study. Six non-DN individuals (patients with renal neoplasm) were recruited as the control group. Their demographic, baseline clinical and biochemical data are summarized in Table 1. No differences were observed between groups regarding gender, age, diastolic BP or haemoglobin values (*P* > 0.05). The systolic BP, and levels of albumin, urea, uric acid, serum creatinine, urine protein and cholesterol were higher in the DN group than in the non-DN group (*P* < 0.05), which indicated the damage inflicted on the kidneys by hyperglycaemia. In particular, the level of protein in the urine was significantly increased in the experimental group compared with the control group (*P* < 0.001), which suggested that podocyte injury occurred in DN patients.

**Table 1:** General and clinical characteristics of patients.

Variable	Non-DN	DN	*P* value
Patients (*n*)	6	31	–
Males/females (*n*)	3/3	19/12	0.140
Age (years)	57.8 ± 3.7	49.9 ± 2.5	0.113
Systolic BP (mmHg)	126 ± 8	145 ± 20	0.033
Diastolic BP (mmHg)	75 ± 5	84 ± 10	0.059
Haemoglobin (g/L)	132 ± 18	115 ± 25	0.098
Albumin (g/L)	41 ± 3	33 ± 7	0.002
Urea (mmol/L)	4.85 ± 0.90	7.75 ± 2.48	<0.001
Uric acid (µmol/L)	294 ± 31	388 ± 15	0.019
Serum creatinine (µmol/L)	70 ± 9	108 ± 8	0.043
Urine protein (g/L)	0.08 ± 0.02	5.40 ± 0.70	<0.001
Cholesterol (mmol/L)	3.69 ± 0.71	5.14 ± 1.25	<0.001

The values are the means ± standard deviation. *P* values for trends in DN and non-DN patients.

Diastolic BP, diastolic blood pressure; DN, diabetic nephropathy; Systolic BP, systolic blood pressure.

### Determination of the podocyte mitochondrial aspect ratio

**Figure 1:**
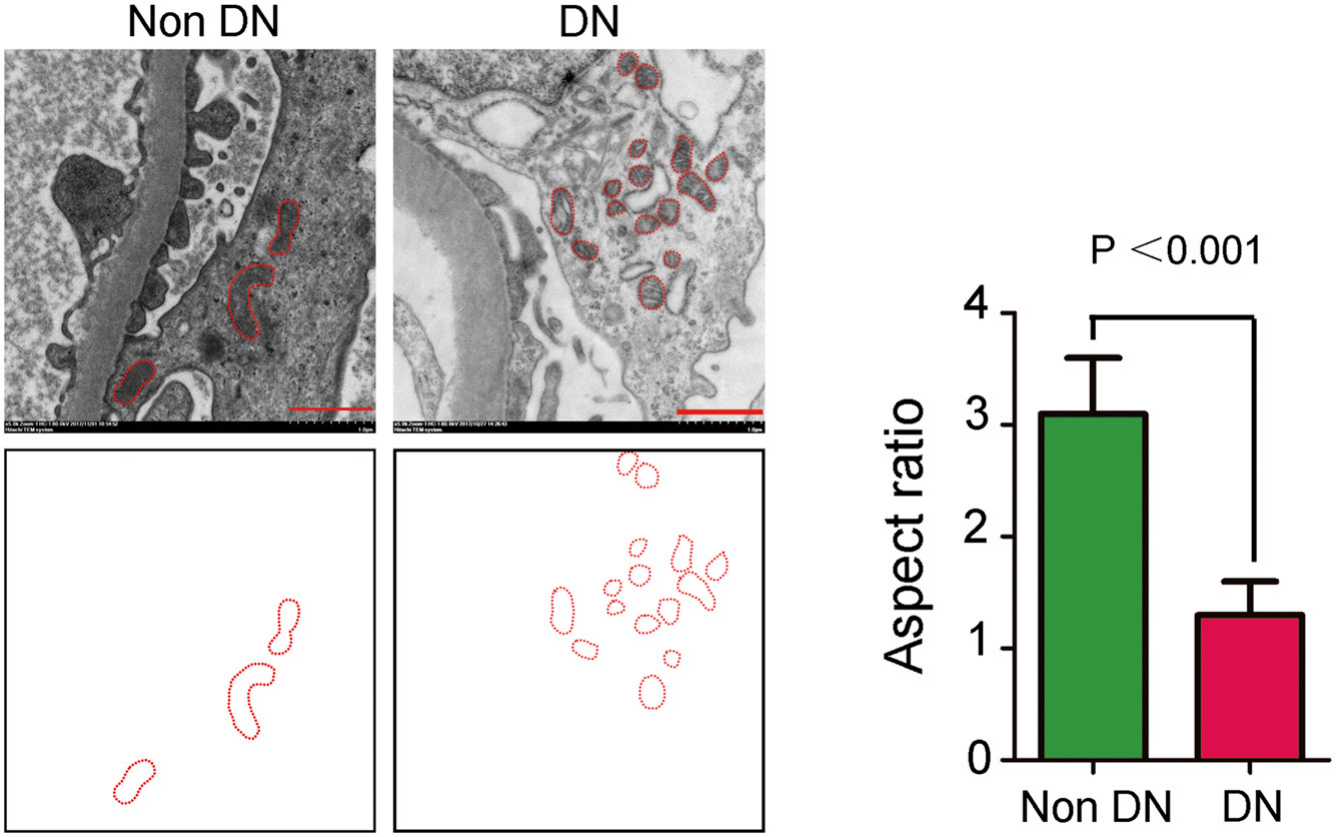
The effect of hyperglycaemia on mitochondrial fragmentation in podocytes. (A) Mitochondrial fragmentation in the glomerular podocytes of DN patients. Representative images from electron microscopy showing elongated mitochondria in the glomerular podocytes of non-DN patients and fragmented mitochondria in the glomerular podocytes of DN patients (original magnification, ×5000). Scale bar, 1 μm.

Renal tissues were obtained from renal biopsies (the experimental group) and nephrectomy (the control group). Ultrastructure examination of the mitochondria in the podocytes from diabetic glomeruli revealed more rounded and circular mitochondria, whereas the mitochondria from the control group were elongated (Fig. 1). The aspect ratio values indicated significant fragmentation of the mitochondria in DN patients (*P* < 0.05, Table 2), suggesting that mitochondrial fission was occurring in the podocytes in DN patients.

**Table 2:** Comparison of mitochondrial aspect ratio in glomerular podocytes between DN and non-DN patients.

Group	*n*	Mitochondrial aspect ratio
Non-DN	6	3.05 ± 0.80
DN	31	1.31 ± 0.54
*P* value		<0.001

Values are means ± standard deviation.* P* values for trends in DN and non-DN patients.

### The podocyte mitochondrial aspect ratio was associated with proteinuria in DN patients

**Figure 2:**
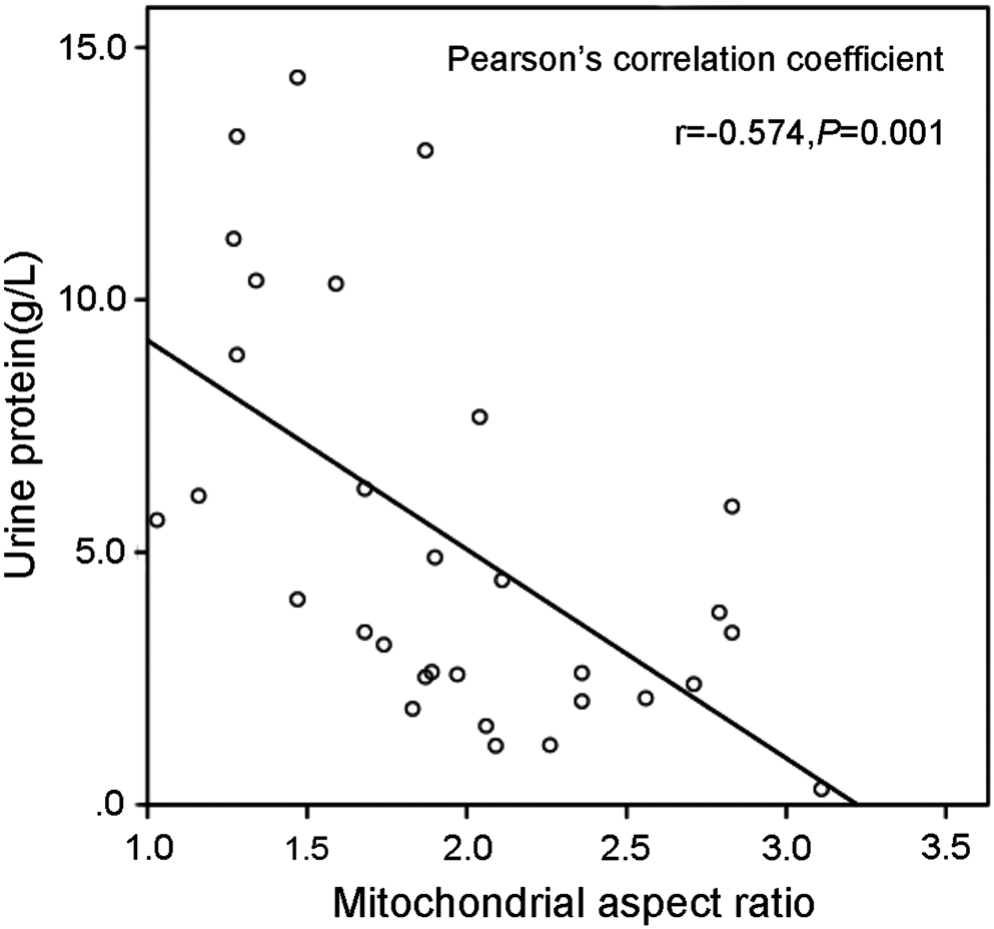
Relationship between the mitochondrial aspect ratio and the urine protein level in DN group. *r*, Pearson’s correlation coefficient.

The relationships between the podocyte mitochondrial aspect ratio values and the clinical parameters are summarized in Table 3. The mitochondrial aspect ratio had a significant positive correlation with haemoglobin values (*r* = 0.407, *P* = 0.023) and a significant negative correlation with urea values (*r* = −0.412, *P* = 0.021). Notably, Fig. 2 shows that the mitochondrial aspect ratio values had a strong negative correlation with the proteinuria levels in DN group (*r* = −0.574, *P* = 0.01). Furthermore, multiple regression analysis verified that the mitochondrial aspect ratio was significantly and independently associated with urine protein levels (β = −0.519, *P* = 0.007).

**Table 3:** Relationship between the clinical characteristics and mitochondrial aspect ratio in the podocytes of patients.

Variable	Pearson’s correlation		Multiple linear regression	
	*R*	*P* value	β	*P* value
Age (years)	−0.224	0.225	−0.201	0.334
Systolic BP (mmHg)	0.139	0.475	0.185	0.356
Diastolic BP (mmHg)	0.136	0.467	−0.189	0.396
Haemoglobin (g/L)	0.407	0.023	−0.143	0.888
Albumin (g/L)	0.287	0.117	−0.054	0.815
Urea (mmol/L)	−0.412	0.021	−0.214	0.420
Uric acid (µmol/L)	−0.252	0.172	−0.206	0.288
Serum creatinine (µmol/L)	−0.172	0.354	−0.119	0.611
Urine protein (g/L)	−0.574	0.001	−0.519	0.007
Cholesterol (mmol/L)	−0.247	0.181	−0.316	0.089

*r*, Pearson’s correlation coefficient; β, standardized coefficient.

### Renal AKAP1 and Drp1 expression in DN patients

**Figure 3:**
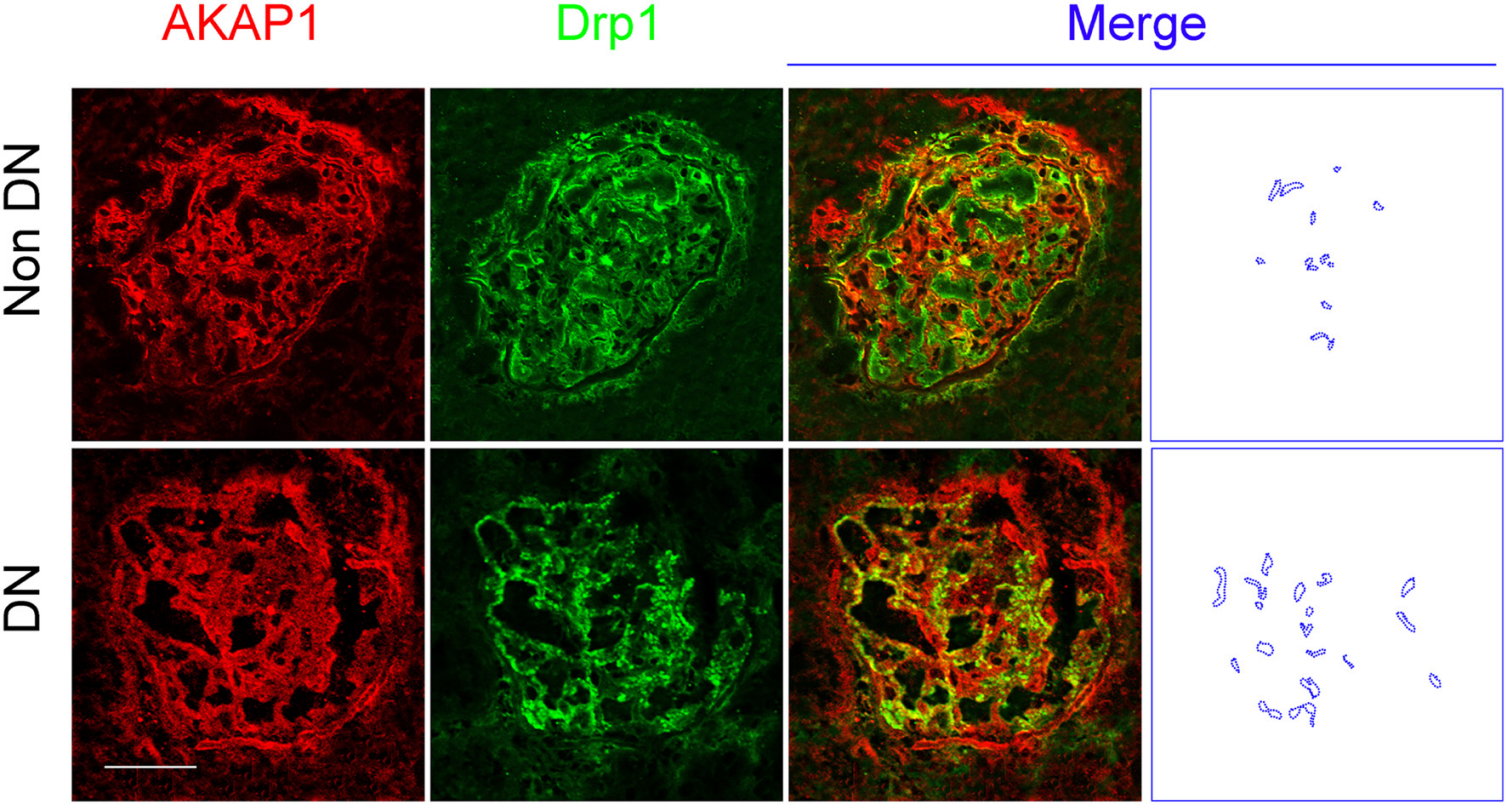
Expression of AKAP1 and Drp1 in the glomeruli of patients. Double immunofluorescence staining of glomerular AKAP1 and Drp1 in the different groups (original magnification, ×400). Scale bar, 20 μm.

Drp1 is considered a key molecule in mitochondrial dynamics. Akap1 is located in the mitochondrial outer membrane and is closely related to mitochondrial function. As shown in Fig. 3, confocal microscopy revealed that AKAP1-Drp1 colocalization was increased in the glomeruli of DN patients compared with control patients. These findings indicated that proteins important in mitochondrial dynamics were involved in the DN process and podocyte injury, which was consistent with our previous studies that showed that mitochondrial fission occurs in podocytes from DN patients.

## Discussion

Mitochondria are called the ‘powerhouses’ of the cell; they also produce the greatest amount of ROS in cells (17). After stimulation with high glucose levels, mitochondria hyperpolarization leads to abnormal ATP synthesis and excessive ROS production, causing cell damage (18). In diabetic animals, increased renal ROS levels are mainly generated by the podocytes (19). In DN patients, urinary exosomes and renal biopsy metabolomics analysis revealed that the expression of multiple regulatory factors associated with mitochondrial metabolism were decreased, such as the mitochondrial biosynthesis factor PGC1α (peroxisome proliferators activated receptor gamma co-activator-1α) (20). These results indicate metabolic abnormalities in the mitochondria. To meet the energy metabolism requirements of tissues and organs, mitochondria may alter their shape and movement. Thus, mitochondria undergo membrane remodelling through cycles of fusion and division and form a dynamic interconnected intracellular network (12, 21). After stimulation with a high level of glucose, the mitochondria in renal tubular cells become short and rounded, and the cristae swell and partly disintegrate (22, 23). Consistent with this observation, our study showed that mitochondria in the podocytes from the glomeruli of DN patients were rounded and circular, whereas mitochondria from non-DN patients were elongated. The mitochondrial aspect ratio analysis indicated significant mitochondrial fission in podocytes from DN patients.

Podocytes have been confirmed to play an important role in the development of the pathological changes that characterize DN, including podocytopenia, hypertrophy, glomerulosclerosis and apoptosis (24). Related to the generation of proteinuria, podocyte injury is an independent risk factor for the progression of DN (4). In this study, we found that the proteinuria level was significantly higher and the mitochondrial aspect ratio value was lower in the DN group than in the non-DN group; the mitochondrial aspect ratio value had a significantly negative correlation with the proteinuria level in DN group. Our observations indicated that hyperglycaemia led to podocyte mitochondrial fission, which caused podocytopenia and proteinuria in DN patients.

Previous studies have confirmed that abnormal mitochondrial dynamics, which cause an imbalance between mitochondrial fission and fusion, are linked to a number of diseases (25, 26). High glucose levels induce the expression of mitochondrial fission proteins (10). The dynamin family is a key component of mitochondrial fission (27). Among the dynamin family members, dynamin-related protein 1 (Drp1) is an important factor. Drp1 is an evolutionarily conserved protein that can self-assemble into large multimeric spirals, mediating mitochondrial fission through GTP-dependent constriction of the mitochondria (28). Under physiological conditions, Drp1 is located in the cytoplasm. When activated by phosphorylation, Drp1 is recruited to the mitochondrial outer membrane, where it promotes mitochondrial division (29, 30). Drp1 phosphorylation during ischaemia/reperfusion induces renal injury, which leads to a reduction in ATP synthesis in the renal tubular cells (31). In the podocytes of diabetic mice, mitochondrial fission is apparently active; deleting podocyte Drp1 results in significantly decreased mitochondrial division, decreased proteinuria and improved podocyte morphology (13). Hence, Drp1 was confirmed to be involved in high glucose level-induced abnormalities in mitochondrial dynamics and podocyte injury. However, the molecular mechanism by which Drp1 regulates these mitochondrial dynamics remains unclear. Furthermore, the role of Drp1 in DN patients has rarely been reported.

Our recent study found that high glucose levels stimulate AKAP1 expression in cultured podocytes (data not shown). AKAP1 is the first member of the A kinase-anchoring protein (AKAP) family. The protein contains a mitochondrial guide peptide sequence, which mediates AKAP1 localization in the mitochondrial outer membrane. In addition, the carboxy terminus of AKAP1 binds mitochondrial ATP synthesis-related messenger RNA (32). In rat hippocampal neuronal cells, DPN, which is an oestrogen receptor hormone agonists, inhibits mitochondrial division via the AKAP1 pathway (33). AKAP1 binds to the mitochondrial outer membrane Na^+^/Ca2^+^ transporter Ncx3, thereby stabilizing mitochondrial calcium flux and alleviating cellular damage caused by hypoxia (34). Deleting AKAP1 increases mitochondrial ROS production and aggravates myocardial infarct size in mouse cardiomyocytes (35). These studies suggest that AKAP1 is involved in the regulation of mitochondrial division in diseases. Our present study demonstrated that AKAP1-Drp1 colocalization was increased in the glomeruli of patients with DN, suggesting that AKAP1 is a candidate for involvement in the regulation of the function of Drp1 to further mediate mitochondrial fission during the pathogenesis of DN. However, due to the limited number of clinical biopsy specimens, more mechanistic studies of mitochondrial dynamics have not been carried out in DN patients, and the role of the AKAP1-Drp1 pathway in mitochondrial dynamics and podocyte injury needs to be explored in future studies.

In summary, our study showed that abnormal mitochondrial dynamics arise in the podocytes of DN patients. Mitochondrial fragmentation was increased, and mitochondrial aspect ratio values were correlated with the proteinuria levels. Further studies are needed to determine the specific molecular mechanism underlying mitochondrial fission and the function of the AKAP1-Drp1 pathway in the pathogenesis of DN.

## Declaration of interest

The authors declare that there is no conflict of interest that could be perceived as prejudicing the impartiality of the research reported.

## Funding

These studies were supported by grants from the National Science Foundation of China (81800615 to Y M and 81770687 to G D).

## Author contribution statement

Design: Guohua Ding. Conduct/data collection: Zhaowei Chen, Yu Tao, Jili Zhu, Yiqiong Ma, Hongxia Yang. Analysis: Yiqiong Ma, Zhaowei Chen, Wei Liang. Writing manuscript: Yiqiong Ma, Guohua Ding.
